# Huntington's like conditions in China, A review of published Chinese cases

**DOI:** 10.1371/currents.RRN1302

**Published:** 2012-02-15

**Authors:** Zhenzhen Zheng, Jean-Marc Burgunder, Huifang Shang, Xiaoyan Guo

**Affiliations:** ^*^Department of Neurology,West China Hospital, Sichuan University, Chengdu Wai Nan Guo Xue Xiang 37# Sichuan, P.R.China and ^†^Prof. M.D. Department of Neurology, University of Bern, Switzerland and University of Sichuan, Chengdu, China

## Abstract

Background: Knowledge about HD in China is lacking in the international literature. We have therefore analyzed the Chinese literature to thoroughly explore the clinical characteristics of Huntington disease in China.

Methods: A computer-based online search of China National Knowledge Infrastructure was performed to review case reports concerning HD published between January 1980 and April of 2011, and the clinical characteristics were extracted.

Results: A total of 92 studies involving 279 patients (157 males and 122 females) were collected, 82.0% of which were from provinces of North China. Most of the cases (97.8%) had a family history of HD, and paternal inheritance (65.5%) was higher than maternal inheritance (34.5%). Onset age was 35.8 (± 11.8) years, death occurred with 45.6 (± 13.5) years after a course of 11.6 (± 5.6) years. Involuntary movements were the most frequent reported presentation (found in 52.3%, including 64.4% in the entire body, 19.8% in the upper limbs, and 13.7% in the head and face). Psychiatric symptoms at onset were reported in 16.1%, and cognitive impairment in 1.8%. With disease progression, 99.6% of patients had abnormal movements, 67.9% cognitive impairment, and 35.0% suffered psychiatric symptoms. Of the reported patients, only 22 underwent IT15 gene testing with positive results.

Conclusion: HD is a well-reported entity in Chinese medical literature, however, only a small number of instances have been proven by molecular diagnosis. Most of the features resemble what is known in other countries. The highly predominant motor presentation, and the higher male prevalence as well as the apparent concentration in Northern China may be due to observational bias. There is therefore a need to prospectively examine cohorts of patients with appropriate comprehensive assessment tools including genetic testing.

## **Introduction**

Huntington disease (HD) has been mainly reported in the West and, to date, knowledge on HD in China is very sparse. It has been claimed, that the incidence is very low, however, well-conducted epidemiological studies are lacking. Furthermore, the phenotype in HD patients has not been well characterized, and it may well be that it is influenced by ethnic background. We have therefore performed an analysis of the literature on HD China by systematically retrieving appropriate reports to describe the clinical manifestations of the disease in this country.  

## **Methods**

Items including Huntington’s disease, Chinese equivalents of the terms: hereditary chorea, chronic progressive chorea, and hysterical chorea were used as keywords to search articles published in the China National Knowledge Infrastructure. Pre-indexing did not reveal any systematic evaluation, prospective or retrospective cohort study regarding HD in China. Case series and individual reports published between January 1^st^, 1980 and April 30^th^, 2011 were included, but reviews and experimental studies on HD excluded. Case reports with non-definite clinical diagnosis or repetitive contents were excluded. Information was extracted about family history, involuntary movements, including the region involved, cognitive disturbances, and psychiatric symptoms. Results of imaging and neurophysiological studies, and of genetic testing were included, where available.    

### **Literature inclusion**

A total of 230 articles related to HD were collected; 136 were excluded since they reported identical data and basic studies; 94 studies involving 547 patients were selected (Table 1). Of these, 306 cases ad to be excluded due to incomplete clinical data. In total, 279 patients were included (157 males and 122 females), with an age of onset of 6–70 years. Of these cases, 236 cases (96.8%) included a family history. A total of 89 patients were noted to have come from a precise region, 16 were from the South, and the remainder were from the North (82.0%), including 15 from Henan Province, 14 from Shandong Province, and 8 from Hebei Province.

Table 1. Reports included in the present survey    

**Table:** 

Year of publication	First author	Male		Female		Molecular testing	Ref.
		N	Age at onset	N	Age at onset		
1980	Wancong Gao	0	na	1	na	na	[11]
1980	Linde Liu	1	42	1	27	na	[12]
1980	Wenshi Li	2	37 to 42	1	54	na	[13]
1980	Xiongya Wu	1	51	1	47	na	[14]
1983	Qian Xu	5	15 to 44	3	7 to 8	na	[15]
1984	Zian Chen	4	32 to 39	1	32	na	[16]
1985	Xiaolian Du	2	5 to 40	3	16 to 19	na	[17]
1985	Jixue Feng	0	na	1	41	na	[18]
1985	Shuyou Fang	4	28 to 46	1	24	na	[19]
1986	Guiqing Wang	2	28 to 58	1	28 to 58	na	[20]
1986	Honglin Fu	0	na	3	34	na	[21]
1986	Hua Shao	1	39	0	na	na	[22]
1987	Wenjun Chen	1	31	0	na	na	[23]
1988	Shicheng Pei	1	43	0	na	na	[24]
1988	Shuqiang Bu	0	na	2	34 to 37	na	[25]
1988	Keqing Ding	3	41 to 55	1	45	na	[26]
1988	Zhiyuan Ha	1	28	1	30	na	[27]
1989	Yongqian Xing	5	20 to 35	3	25 to 43	na	[28]
1989	Xuesong Tu	0	na	1	na	na	[29]
1989	Fuyuan Shao	1	7	0	na	na	[30]
1990	Yuxiang Xu	4	17 to 42	1	35	na	[31]
1990	Chuandong Wu	2	17 to 36	0	na	na	[32]
1990	Jiaxi Huang	0	na	4	34 to 40	na	[33]
1991	Zhaoxiang Zeng	0	na	1	31	na	[34]
1992	Ke Fan	4	27 to 38	2	38 to 56	na	[35]
1992	Changdao Sun	3	24 to 30	2	21 to 25	na	[36]
1992	Chengyu Chen	4	27 to 47	0	na	na	[37]
1993	Fengying Hou	1	47	0	na	na	[38]
1994	Yanjun Gao	1	48	0	na	na	[39]
1994	Qishan Dong	1	42	1	38	na	[40]
1994	Yuejin Huang	0	na	1	42	na	[41]
1995	Xiaomei Yao	0	na	2	46 to 47	na	[42]
1995	Xiuhua Fan	1	47	0	na	na	[43]
1995	Chenghao Chu	4	10 to 65	1	20	na	[44]
1995	Jiying Wang	0	na	1	32	na	[45]
1995	Ronghua Yong	1	60	1	48	na	[46]
1996	Kun Liu	0	na	1	42	na	[47]
1996	Zhaozhong Shen	2	na	0	na	na	[48]
1997	Meiju Hou	1	36	0	na	na	[49]
1997	Jiaming Xia	3	18 to 35	2	18	na	[50]
1997	Wenhua Sun	0	na	1	25	na	[51]
1998	Xiangming Fang	5	18 to 40	0	na	na	[52]
1998	Weizhou Liu	1	23	0	na	na	[53]
1998	Yujin Zhang	6	24 to 50	4	30 to 55	na	[54]
1998	Xiaoping Yang	2	30 to 40	2	13 to 33	na	[55]
1998	Kai Feng	1	56	0	na	na	[56]
1998	Zuozhi Gao	2	33 to 54	1	34	na	[57]
1998	Xiaoping Zeng	3	43 to 54	1	45	na	[58]
1998	Yuchen Sun	1	42	2	45 to 46	na	[59]
1999	Weiguo Yang	0	na	1	39	na	[60]
1999	Guilan Wang	0	na	1	8	na	[61]
1999	Bin Liu	4	32 to 45	3	34 to 38	na	[62]
2001	Xi Zhang	1	50	0	na	na	[63]
2001	Jing Chen	1	24	0	na	na	[64]
2001	Wen Li	2	21 to 26	3	25 to 26	na	[65]
2001	Qinglin Dong	4	23 to 36	2	30 to 32	na	[66]
2001	Huizhi Fan	0	na	1	32	na	[67]
2001	Liansheng Xu	5	29 to 54	5	32 5o 52	na	[68]
2002	Jing Liu	3	28 to 46	5	35 to 50	na	[69]
2002	Benqiang Deng	1	66	0	na	na	[70]
2002	Ye Tian	1	70	1	40	na	[71]
2003	Liqun Fang	1	31	0	na	na	[72]
2003	Huize Ma	2	31 to 52	3	33 to 45	na	[73]
2003	Yuhai Zhao	2	22 to 40	1	30	N CAG repeats	[74]
2004	Feng Tian	1	54	2	38 to 50	na	[75]
2004	Mingbing Chen	1	32	0	na	na	[76]
2004	Weiwei Dong	1	14	0	na	na	[1]
2004	Jun Chen	2	52	2	44 to 54	na	[76]
2004	Jiamu Wu	2	31 to 33	0	na	na	[77]
2004	Beilei Zhu	1	28	0	na	Genetic test (no N CAG repeats)	[78]
2005	Liyan Guo	0	na	1	32	na	[79]
2006	Shiyong Zhang	0	na	1	52	na	[80]
2006	Huamei Wang	2	41 to 48	0	na	na	[81]
2006	Fang Lin	4	421 to 58	0	na	na	[82]
2006	Baorong Zhang	3	30 to 45	5	18 to 36	N CAG repeat	[83]
2006	Zhilin Shi	2	33 to 45	1	45	Genetic test (no N CAG repeats)	[84]
2007	Yuan Liu	3	33	1	17	N CAG repeats	[85]
2007	Yanchun Geng	2	28 to 29	0	na	na	[86]
2008	Wei Xu	3	25 to 36	2	27 to 45	Genetic test (no N CAG repeats)	[87]
2008	Jin Yu	1	41	4	32 to 45	na	[88]
2008	Ning Wang	1	na	2	na	N CAG repeat	[89]
2008	Zhouri Li	2	40 to 43	1	41	na	[90]
2008	Yiming Feng	1	33	0	na	na	[91]
2009	Xingwang Song	2	41 to 57	4	6 to 58	N CAG repeat	[92]
2009	Qiuhong Zheng	2	50 to 60	3	41 to 60	na	[93]
2009	Meiying Cai	1	30	2	35 to 45	N CAG repeat	[94]
2009	Weijie Liu	0	na	1	37	na	[95]
2010	Meihua Zhu	0	na	1	35	na	[96]
2010	Ge Gao	3	36 to 42	0	na	N CAG repeat	[97]
2010	Jing Ma	0	na	1	46	na	[98]
2011	Min Li	0	na	1	51	na	[99]
	Total	157		122			

     Most (65%) of the patients had onset in middle age (Table2), with a mean of 35.8 years (± 11.8), mean age at death was 45.6 years (± 13.5, range 13–69), and mean course from onset to death was of 11.6 years (± 5.6, range 3–30). Around 9 % had a juvenile onset. The study included 115 families. Paternal inheritance was more often found than maternal inheritance (Table 2), and age of onset with paternal inheritance was lower than maternal inheritance (34 ± 10 versus 37 ± 10 years,* P* < 0.05). The age at death (46 ± 15 *vs*. 50 ± 10 years), and the course of disease (12 ± 6 *vs*. 12 ± 4 years), were not significantly different. In addition, no difference was found between male and female patients in terms of age of onset, death age (47 ± 14 versus 46 ± 12 years), and course of disease (12 ± 6.0 *vs*. 10.7 ± 5.0 years). Within the cohort, 55 patients death were reported, of which three were due to suicide. 

 Table 2. Clinical features of the reported patients  

**Table:** 

	N of total with available data	%
Inheritance		
Paternal	127/194	65.5
Maternal	67/194	34.5
Anticipation	42/59	71.2
Age at onset (yr)		
< 20	24/279	8.6
30–55	182/279	65.2
Presentation at onset		
Abnormal movements	146/196	52.3
Generalized	94/146	64.4
Head and face	20/146	13.7
Upper limbs	29/146	19.8
Lower limbs	3/146	2.1
Psychiatric disorder	45/196	16.1
Cognitive impairment	5/196	1.8
Course		
Abnormal movements	242/243	99.6
Psychiatric disorder	85/243	35.0
Cognitive impairment	165/243	67.9

   The most frequent presentation at onset were abnormal movement found in more than half of the patients (Table 2), with a predilection for the face and upper limbs when it was not generalized. In the course of the disorder most patients developed abnormal movements, followed by psychiatric symptoms and cognitive impairment (Table 2). There were some specific features in single patients, for example, one displayed speech impairment, instability of gait and cognitive impairment, with no obvious involuntary movement [1]. Dysarthria and dysphagia was also reported during the course in 26 % of the patients. One single patient had epilepsy at onset.

### **Laboratory investigations**

     In a total of 48 patients with electroencephalograms, 34 (70.8%) had abnormal curves, mostly with mild slowing. In a total of 16 patients undergoing cerebrospinal fluid examination, three (18.8%) displayed abnormally increased protein levels. In a total of 89 patients undergoing cranial imaging, 80 (90.0%) presented with abnormalities of varying extent, including 65 (73.0%) with brain atrophy and lateral ventriculomegaly, 2 (2.2%) with selective caudate nucleus atrophy, and 26 (29.2%) with brain atrophy, lateral ventriculomegaly, and caudate nucleus atrophy. * IT15 *gene detection was reported positive in 38 patients, of which CAG repeats were clearly reported in 33 of the cases. The number of CAG repeats was greater in patients with a younger age of onset (mean of 61 in patients with onset before versus 46 with age of onset after 30).   

## **Discussion**

The prevalence of HD is quite variable, with figures varying between 0.5 (Finland) [2], 1 (Croatia [3]) and 10 (German speaking European countries [4]) per 100 000 in Europe, and with high local prevalence in some communities, like in Venezuela (almost 700 in the Lake Maracaibo region [5]. It is usually thought that the prevalence in Asia is lower, however, fewer data than in the West have been reported so far. In Japan reported estimations ranged between 0.1 [6] and 0.7 [7]. Earlier estimation in Hongkong are within the same range [8], however, no data have been so far reported for mainland China. The present survey of Chinese literature on HD shows that the disease is indeed present in this country, but does not provide precise clues on the prevalence of the disorder. It also suggests a higher prevalence in Northern China, however this may also be a report bias. The number of juvenile cases reported seems higher than in other regions of the world and there is a male predominance in overall prevalence. However, the mean age of onset is otherwise consistent, but the course seems shorter with absence of the gender difference reported earlier [9]. The majority of patients had a positive family history, and only five cases were determined to be sporadic. Moreover, the number of cases due to paternal inheritance was significantly greater than that from maternal inheritance, with a significantly younger age of onset with paternal inheritance, which was in accordance with previously published results. Only one case among the 20 with juvenile onset was reported to have epileptic seizure, which is in contrast to the literature, reporting up to 30% of them. Studies have suggested that the suicide rate of HD patients is significantly greater than healthy individuals, in particular in early or advanced stages [10]. Only three patients were reported to have committed suicide, however, data regarding suicide in China are not available for comparison. In general the other aspects were similar to the reports in other populations. However, only a small number had a molecular confirmed diagnosis, but the trend to earlier age of onset with higher triplet repeat numbers is found here also. 

     In conclusion, ethnic differences among Chinese with HD as compared to other populations are possible. However, the use of appropriate clinical assessment tools and molecular genetic testing in a larger cohort of patients is urgently needed. For this reason a Chinese Huntington’s disease network is going to be launched.   

## Competing interests

      The authors have declared that no competing interests exist

## References

[ref-2524917065] Dong, W., J. Zhang, and S. Lang, The functional disorder analysis of a case with Huntington’s disease. Zhongguo Linchuang Kangfu, 2004. 10: p. 1919.

[ref-2570778026] Palo J, Somer H, Ikonen E, Karila L, Peltonen L. Low prevalence of Huntington's disease in Finland. Lancet. 1987 Oct 3;2(8562):805-6. 2889026 10.1016/s0140-6736(87)92545-1

[ref-964885372] Hecimovic, S., et al., Genetic background of Huntington disease in Croatia: Molecular analysis of CAG, CCG, and Delta2642 (E2642del) polymorphisms. Hum Mutat, 2002. 20(3): p. 233 10.1002/humu.9055 12204002

[ref-146022288] Laccone, F., et al., DNA analysis of Huntington's disease: five years of experience in Germany, Austria, and Switzerland. Neurology, 1999. 53(4): p. 801-6. 10.1212/wnl.53.4.801 10489044

[ref-897322579] Avila-Giron, R., Medical and Social Aspects of Huntington's chorea in the state of Zulia, Venezuela. Adv Neurol, 1973. 1: p. 261-266.

[ref-1505446255] Kanazawa, I., et al., Studies on DNA markers (D4S10 and D4S43/S127) genetically linked to Huntington's disease in Japanese families. Hum Genet, 1990. 85(3): p. 257-60. 10.1007/BF00206741 1975553

[ref-4002592542] Nakashima, K., et al., Epidemiological and genetic studies of Huntington's disease in the San-in area of Japan. Neuroepidemiology, 1996. 15(3): p. 126-31. 10.1159/000109899 8700304

[ref-1255473940] Leung, C.M., et al., Huntington's disease in Chinese: a hypothesis of its origin. J Neurol Neurosurg Psychiatry, 1992. 55(8): p. 681-4. 10.1136/jnnp.55.8.681 PMC489204 1388199

[ref-3785319020] Foroud, T., et al., Differences in duration of Huntington's disease based on age at onset. J Neurol Neurosurg Psychiatry, 1999. 66(1): p. 52-6. 10.1136/jnnp.66.1.52 PMC1736160 9886451

[ref-580344739] Baliko, L., B. Csala, and J. Czopf, Suicide in Hungarian Huntington's disease patients. Neuroepidemiology, 2004. 23(5): p. 258-60. 10.1159/000079953 15316254

[ref-1046804543] Gao, W., A case of Huntington’s disease. Bengbu Yixueyuan Xuebao, 1980. 1: p. 66-67.

[ref-426118220] Liu, L., Y. Xu, and B. Liu, Two cases of Huntington’s disease. Zhongguo Shenjing Jingshen Jibing Zazhi, 1980. 2: p. 128.

[ref-1188623259] Li, W. and S. Luan, The survey of one family with Huntington’s disease. Zhongguo Shenjing Jingshen Jibing Zazhi, 1980. 6(5): p. 292-295.

[ref-1963005097] Wu, X., Two cases of Huntington’s disease. Shaanxi Yixue Zazhi, 1983. 12(5): p. 41-42.

[ref-185620348] Xu, Q., J. Qi, and R. Han, A family survey of Huntington’s disease. Harbin Yiyao, 1983 2: p. 75-77.

[ref-1492337928] Chen, Z., Two cases of Huntington’s disease with lithium carbonate treatment. Tianjin Yiyao, 1984 4: p. 242-243.

[ref-2675103732] Du, X. and X. Cui, A case report of Huntington’s disease. Ningxia Yixueyuan Xuebao, 1985. 3: p. 15-16.

[ref-3520198248] Feng, J., A case report of Huntington’s disease-with family survey. Zhongguo Shiyong Neike Zazhi, 1985. 5(7): p. 397.

[ref-3256360752] Fang, S., et al., A four generation family of Huntington’s disease. Zhengzhou Daxue Xuebao: Yixue Ban, 1985. 20(4): p. 308-309.

[ref-2901946024] Wang, G., et al., An analysis of four families with Huntington’s disease. Shanghai Yixue, 1986. 9(2): p. 90-92.

[ref-1768441905] Fu, H., et al., A survey and assessment of amino acids level in serum of three families with Huntington’s disease. Jiangxi Yixueyuan Xuebao, 1986. 4: p. 83-87.

[ref-3965275347] Shao, H., A case report of Huntington’s disease. Xin Yixue, 1986. 17(2): p. 77-78.

[ref-3713342616] Chen, W. and D. Xu, An analysis of a family with Huntington’s disease. Huanan Guofang Yixue Zazhi, 1987. 3: p. 91-92.

[ref-2322541101] Pei, S., A case report and family survey of Huntington’s disease. Lanzhou Daxue Xuebao: Yixue Ban, 1988. 1: p. 56-57.

[ref-577962357] Bu, S. and H. Hou, A report of two families with Huntington’s disease. Jiangsu Yiyao, 1988. 4: p. 226-227.

[ref-472750831] Ding, K. and W. Wang, Following up study of patients with IT15 gene carriers by EEG. Huazhong Shifan Daxue Xuebao: Ziran Kexue Ban, 1988. 22(3): p. 363-365.

[ref-3520409497] Ha, Z., A family report of Huntington’s disease with abnormal chromosome. Zhongguo Shenjing Jingshen Jibing Zazhi, 1988. 2: p. 82.

[ref-945099995] Xing , Y., Z. Wu, and F. Chen, Five cases of Huntington’s disease. Guangdong Yixueyuan Xuebao, 1989. 4: p. 350-351.

[ref-1441501239] Tu, X., A case report of Huntington’s disease. Linchuang Neike Zazhi, 1989. 6(20): p. 111.

[ref-657669637] Shao, F., C. Zai, and L. Dou, A case report of Huntington’s disease presenting rigidity. Zhongguo Shenjing Jingshen Jibing Zazhi, 1989. 2: p. 114.

[ref-4231286099] Xu, Y. and M. Liu, Three cases of Huntington’s disease. Weifang Yixueyuan Xuebao, 1990. 12(4): p. 72-73.

[ref-129462184] Wu, C., A case report of patient with Huntington’s disease with initial symptom of mental disorder. Linchuang Shenjingbingxue Zazhi, 1990. 2: p. 125-126.

[ref-834857684] Huang, J., Four cases of Huntington’s disease in one family. Zhongguo Yousheng yu Yichuan Zazhi, 1990. 4: p. 89-90.

[ref-3736207284] Zeng, Z. and J. Zhang, A family survey of Huntington’s disease. Zhongguo Yejin Gongye Yixue Zazhi, 1991. 8(1): p. 43.

[ref-3527715928] Fan, K., T. Zhao, and L. Feng, Twelve cases of Huntington’s disease in one family. Linchuang Shenjingbingxue Zazhi, 1992. 5(3): p. 176-177.

[ref-1361923140] Sun, C., et al., A family survey of Huntington’s disease. Zhongguo Yousheng yu Yichuan Zazhi, 1992. 4: p. 105-107.

[ref-1111889778] Chen, C., et al., Four cases of Huntington’s disease with ventrolateral nuclei of thalamus destruction treatment. Zhongguo Shenjing Jingshen Jibing Zazhi, 1992(4): p. 256.

[ref-1337597950] Hou, F. and G. Li, A family survey of Huntington’s disease. Jinzhou Yixueyuan Xuebao, 1993. 14(2): p. 82.

[ref-2818592113] Gao, Y., et al., The clinic feature of a family with Huntington’s disease. Nao yu Shenjing Jibing Zazhi, 1994. 3: p. 184.

[ref-2104750889] Dong, Q., Huntington’s disease with haloperidol andγ- aminobutyric acid treatment. Zhongguo Yousheng yu Yichuan Zazhi, 1994. 2(4): p. 118.

[ref-2694822854] Huang, Y., Five cases of Huntington’s disease in one family. Zhongfeng yu Shenjing Jibing Zazhi, 1994. 5(304).

[ref-165452473] Yao, X., S. Shen, and J. Wang, Two cases with Huntington’s disease. Tianjin Yiyao, 1995. 23(10): p. 605-606.

[ref-4071333552] Fan, X. and H. Zhang, A case with Huntington’s disease associated with paranoid psychosis. Zhongguo Shenjing Jingshen Jibing Zazhi, 1995. 21(5): p. 318.

[ref-3914278448] Chu, C., Nine cases of Huntington’s disease in one family. Zhongfeng yu Shenjing Jibing Zazhi, 1995. 12(2): p. 108-109.

[ref-1511932677] Wang, J., et al., A case with Huntington’s disease. Taishan Yixueyuan Xuebao, 1995. 16(1): p. 61.

[ref-3507549914] Yong, R. and M. Yang, Two families of Huntington’s disease. Zhongguo Yousheng yu Yichuan Zazhi, 1995. 3(6): p. 109.

[ref-1442276519] Liu, K., L. Xu, and T. Wang, A case of Huntington’s disease and the family survey report. Zhongguo Zonghe Linchuang, 1996. 12(5): p. 255-256.

[ref-431705654] Shen, Z., et al., A study of D4S（125）linked polymorphism associated with Huntington’s disease. Shanghai Yixue, 1996. 19(1): p. 5-7.

[ref-486891569] Shen, Z., et al., A study of D4S（125）linked polymorphism associated with Huntington’s disease. Shanghai Yixue, 1996. 19(1): p. 5-7.

[ref-1275446189] Xia, J. and Y. Zhou, Ten cases of Huntington’s disease in two families. Anhui Yixue, 1997. 18(3): p. 64-65.

[ref-4219975259] Sun, W. and C. Zhang, Eight cases of Huntington’s disease in one family. Linchuang Huicui, 1997. 12(8): p. 356-357.

[ref-3921550393] Fang, X., Two families of Huntington’s disease. Shiyong Yixue Zazhi, 1998. 14(4): p. 342.

[ref-962209450] Liu, W., H. Chen, and H. Pan, The family survey of Huntington’s disease. Zhongguo Jiceng Yiyao, 1998. 5(6): p. 332.

[ref-2099551473] Zhang, Y., Clinical analysis and genetic counseling of 13 cases with Huntington’s disease. Linchuang Yixue, 1998. 18(2): p. 19-20.

[ref-1888517549] Yang, X. and Y. Xu, Five cases of Huntington’s disease in one family. Sichuan Yixue, 1998. 19(4): p. 354-355.

[ref-3252481735] Feng, K., X. Lv, and J. Liu, A family report of Huntington’s disease. Zhongguo Meitan Gongye Yixue Zazhi, 1998. 1(2): p. 98.

[ref-4107546331] Gao, Z., X. Yang, and B. Tang, A survey of three families with Huntington’s disease. Zhongguo Yishi Zazhi, 1998. 3(1): p. 43-44.

[ref-700223663] Zeng, X., Four cases of Huntington’s disease in one family. Zhonghua Yixue Yichuanxue Zazhi, 1998. 15(4): p. 217.

[ref-3041678919] Sun, Y., Huntington’s disease. Tianjin Yiyao, 1998. 26(1): p. 38-39.

[ref-808531999] Yang, W., Five cases of Huntington’s disease in one family. Linchuang Neike Zazhi, 1999. 16(3): p. 156.

[ref-1111092004] Wang, G., A child case with Huntington’s disease. Chengde Yixueyuan Xuebao, 1999. 16(2): p. 183-184.

[ref-930670673] Liu, B., Seven cases of Huntington’s disease in one family. Zhonghua Yixue Yichuanxue Zazhi, 1999. 16(2): p. 73.

[ref-1813956066] Zhang, X., A case with mental disorder associated with Huntington’s disease. Linchuang Jingshen Yixue Zazhi, 2001. 1(5): p. 285.

[ref-3050225771] Chen, J. and T. Zou, A case of Huntington’s disease misdiagnosed as schizophrenia. Guiyang Yixueyuan Xuebao, 2001. 26(6): p. 557-560.

[ref-1713818247] Li, W., Five cases of Huntington’s disease in one family. Nao yu Shenjing Jibing Zazhi, 2001. 9(5): p. 314.

[ref-3597223271] Dong, Q. and Z. Cheng, Analysis of seven cases of Huntington’s disease in one family. Linchuang Shenjingbingxue Zazhi, 2001. 14(3): p. 175.

[ref-666041106] Fan, H., A family survey of Huntington’s disease. Zhongguo Shengyu Jiankang Zazhi, 2001. 12(4): p. 156.

[ref-84952942] Xu, L. and F. Wang, Fifteen cases of Huntington’s disease in one family. Xiandai Kangfu, 2001. 5(1): p. 92.

[ref-1450994747] Liu, J., J. Han, and J. Wang, Two families of Huntington’s disease. Zhongfeng yu Shenjing Jibing Zazhi, 2002. 19(6): p. 374.

[ref-3519424995] Deng, B., et al., Eleven cases of Huntington’s disease in one family. Zhonghua Yixue Yichuanxue Zazhi, 2002. 19(2): p. 137.

[ref-1630850833] Tian, F., Pedigree analysis of a series of Huntington’s disease. Zhongguo Minzheng Yixue Zazhi, 2002. 14(4): p. 237-238.

[ref-1737960849] Fang , L., A case of mental disorder associated with Huntington’s disease. Linchuang Jingshen Yixue Zazhi, 2003. 13(6): p. 330.

[ref-766402950] Ma, H., L. Li, and Y. Wang, The clinical features of five families with Huntington’s disease and literature review. Zhongguo Kangfu Lilun yu Shijian, 2003. 9(7): p. 434-436.

[ref-2404485412] Zhao, Y., et al., IT15 gene analysis of two families with Huntington’s disease from Liaoning province. Zhongguo Yike Daxue Xuebao, 2003. 32(4): p. 355-357.

[ref-1178694636] Tian , Y., Three cases of Huntington’s disease in one family. Hanshao Jibing Zazh, 2004. 11(1): p. 60.

[ref-2001379017] Chen, M., et al., A case of Huntington’s disease undergoing emergency anesthesia. Zhonghua Mazuixue Zazhi., 2004. 24(10): p. 798.

[ref-426929820] Wu, J., A family of Huntington’s disease. Zhonghua Yixue Yichuanxue Zazhi, 2004. 21(2): p. 178.

[ref-45580129] Zhu, B., et al., Clinical features and gene study of five cases with Huntington’s disease in one family. Wenzhou Yixueyuan Xuebao, 2004. 34(4): p. 307-308.

[ref-372500488] Guo, L. and Y. Li, A case of Huntington’s disease. Xiandai Yiyao Weisheng, 2005. 21(3): p. 1648.

[ref-2500780941] Zhang, S., Y. Li, and H. Guo, A family of Huntington’s disease. Zhonghua Yixue Yichuanxue Zazhi, 2006. 23(5): p. 535.

[ref-631457270] Wang, H., Q. Zhou, and J. Wu, A family of Huntington’s disease. Shenjing Jibing yu Jingshen Weisheng, 2006. 6(5): p. 383.

[ref-2913934466] Lin, F., Z. Chen, and C. Liu, Four cases of Huntington’s disease in one family. Zhonghua Yixue Yichuanxue Zazhi, 2006. 23(4): p. 486.

[ref-837468433] Zhang, B., et al., IT15 gene study of two families with Huntington’s disease. Yichuan, 2006. 28(11): p. 1345-1349. 10.1360/yc-006-1345 17098699

[ref-140772958] Shi, Z., et al., The clinical features analysis and gene diagnosis of a family with Huntington’s disease. Zhongguo Shengyu Jiankang Zazhi, 2006. 17(2): p. 99-100.

[ref-1444209463] Liu, Y., et al., Intergeneration CAG expansion in a Wuhan juvenile-onset Huntington disease family. Neuroscience Bull, 2007. 23(4): p. 198-202. 10.1007/s12264-007-0029-7 PMC5550581 17687393

[ref-2298680351] Geng, Y., Five cases of Huntington’s disease in one family. Shiyong Quanke Yixue, 2007. 5(11): p. 106.

[ref-1122917166] Xu, W., et al., IT15 gene study in a family with Huntington’s disease. Zhongguo Xiandai Yixue Zazhi, 2008. 18(19): p. 2832-2834.

[ref-3437981768] Yu, J., et al., Two cases with Huntington’s disease. Wujing Yixue, 2008. 19(8): p. 756-757.

[ref-2935057038] Wang, N. et. al, Genetic diagnosis of Huntington’s disease in two families. Chin J Contemp Neurol Neurosurg, 2008. 8(2): p. 124-125.

[ref-1479063331] Li, Z. et. al, IT 15 genetic diagnosis of Huntington’s disese in one family. China Journal of Modern Medicine, 2008. 18(19): p. 2832.

[ref-4242937726] Feng, Y. et. al, Radiological features of Huntington’s disease. Clinical Radiology, 2008. 10: p. 27.

[ref-1512559547] Song, X. et al, Clinical manifestation of Huntington’s disease and IT 15 mutation characteristics. Practical Medicine, 2009. 20(25): p. 3459-3461.

[ref-1888224440] Zheng, Q. et al, Analysis of clinical features of Huntington’s disease in one family. Clinical focus, 2009(24): p. 14.

[ref-303374294] Cai, M. et al, Diagnostic research of HTT gene in one family of Huntington’s disease. Chin J Lab Med, 2009. 26(6): p. 688-190.

[ref-1061675318] Liu, W. et al, Bilateral deep brain stimulation for Huntington’s disease and literature review. Chinese Journal of Neurosurgery, 2009. 25: p. 7.

[ref-1072689055] Zhu, M. et al, The nursing of Huntington’s disease after deep brain stimulation procedures. Chinese Journal of Clinical Research, 2010. 23(5): p. 428-429.

[ref-2387140583] Gao, G. et al, CAGn test of IT 15 gene in two families of Huntington’s disease. Journal of Zhengzhou University, 2010. 45(6): p. 936-939.

[ref-215617064] Ma, J., Nursing of a patient with chronic progressive chorea and decubital ulcers. Chin J Misdiagn, 2010. 36(10): p. 8914.

[ref-1207212771] Li, M. et al, A case report of Huntington’s disease accompanied by psychiatric disorders. J Clin Psychiatry, 2011. 1(21): p. 48.

